# Reclaiming Ruthenium: A Comprehensive Review of Hydrometallurgical Strategies for Precious Metal Recovery

**DOI:** 10.3390/ma19030461

**Published:** 2026-01-23

**Authors:** Ewa Rudnik

**Affiliations:** Faculty of Non-Ferrous Metals, AGH University of Krakow, Mickiewicz Ave. 30, 30-059 Krakow, Poland; erudnik@agh.edu.pl

**Keywords:** ruthenium, critical metal, primary production, secondary sources, leaching, recycling, catalysts, metallurgical waste, wastewaters, spent nuclear fuel

## Abstract

Ruthenium, a critical metal, plays an increasingly important role in modern applications, such as catalysts for chemical synthesis and the production of hard disk drives. As a result, the supply has struggled to meet the growing demand in recent years. The economic position of ruthenium presents an opportunity to examine the methods of its extraction, particularly given that it is a lesser-known platinum group metal. This article explores the concentration of ruthenium in natural sources and the methods used in primary production, with a particular focus on hydrometallurgical techniques applied at an industrial scale. It also discusses secondary ruthenium-containing materials, including spent catalysts, metallurgical by-products, wastewaters, spent nuclear fuel. The article provides a detailed analysis of the composition of these materials, emphasizing hydrometallurgical methods like leaching and separation processes, along with the recovery of final products.

## 1. Introduction

**Figure 1:**
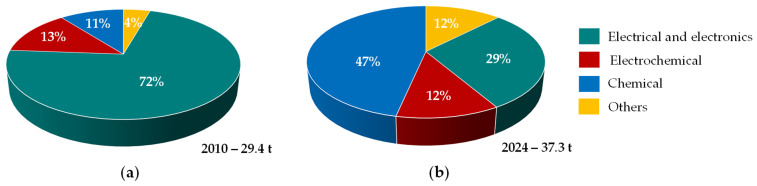
Structure of global ruthenium demand in: (**a**) 2010, (**b**) 2024 (based on [1,2]).

Ruthenium Ru, one of the lesser-known platinum group elements, has gained significance in recent years owing to its growing importance in advanced technologies [1,2]. Its applications (Figure 1), once primarily confined to wear-resistant electrical contacts, catalysts for chemical synthesis, and alloy hardening, are now closely linked to industrial organic synthesis [3], energy transition technologies, and high-performance computing. Ruthenium catalysts for chemical processes are primarily used in China, notably in the expanding caprolactam sector for polyamide PA 6 production, which is widely employed in engineering plastics [1]. Ruthenium and its compounds can play a crucial role in clean energy systems, serving as catalysts in proton exchange membrane electrolysers for hydrogen [4] and oxygen [5] production, in fuel cell technologies [4], and in automotive catalytic converters for the reduction of nitrogen oxide emissions [6]. It is also used as a diffusion barrier in chip resistors and electrical contacts for highly efficient microelectronics [7], as well as in antiferromagnetically coupled media for hard disk drives employed in large-scale data centers supporting artificial intelligence services [1,8]. Owing to its exceptional chemical inertness, corrosion resistance, extreme hardness (2160 MPa), high melting point (2334 °C) [9], and outstanding catalytic properties, ruthenium has virtually no substitutes capable of delivering a comparable combination of performance characteristics.

Market forecasts indicate that the global ruthenium industry is expected to reach approximately 45 t by 2033, with a projected compound annual growth rate of 3.6% [10]. However, data from the most recent years (2023–2024) already reveal a persistent imbalance between primary supply and demand, amounting to approximately 3–4 t per year, with projected shortages of around 8 t in 2025 [1]. This imbalance was temporarily supplemented by producer stocks, as they took advantage of strong demand from the chemical sector and hard disk drive manufacturing in Asia. It also reflected in ruthenium prices, which have shown a steady upward trend since 2024, rising from about 18.5 USD/g in January 2025 to about 50 USD/g by January 2026 [11]. As a result, ruthenium emerged as one of the strongest-performing metals in 2025, reaching its highest price level observed over the past five years. Nevertheless, it remains the least expensive among all platinum group metals.

**Figure 2:**
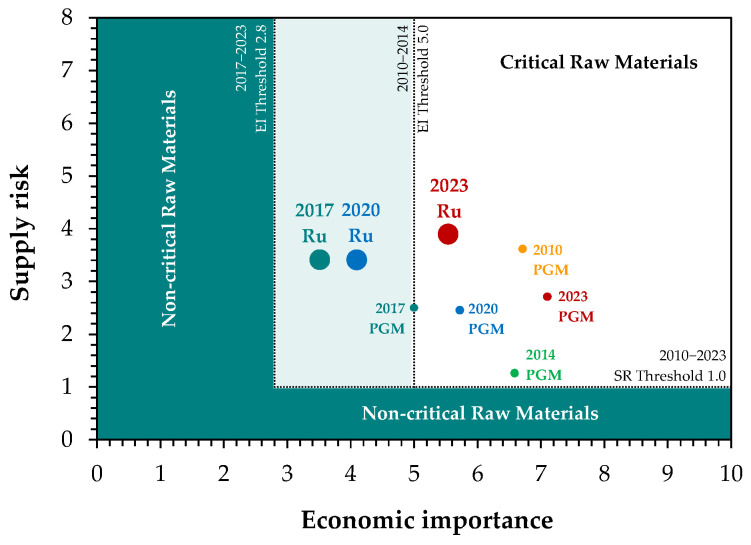
Evolution of ruthenium position in the European Union criticality matrix compared to the platinum group metals PGM as a whole (based on [12]).

Economic importance of ruthenium is furthermore reflected in its classification as a critical and strategic metal by the European Union [12], the United States [13], Canada, and South Africa [14] in their most recent lists. Notably, in the EU, ruthenium has gradually emerged as an element of increasing significance, as reflected in the 2023 list (Figure 2). Although supply risk has risen only modestly, it is primarily driven by the concentration of production in a one dominant region, which introduces a degree of uncertainty into supply chains.

All of these factors clearly highlight ruthenium recycling, particularly given that it is a scarce metal. Although recycling of platinum group metals is generally considered standard practice rather than an exception, the hydrometallurgical aspects of processing secondary ruthenium materials remain particularly relevant in the context of recently observed growth in metal demand. Therefore, the aim of this work is to present advances in this field in relation to ruthenium production from natural raw materials.

## 2. Materials and Methods

The literature review was conducted using keywords relevant to the topic, including ruthenium, (bio)hydrometallurgy, recycling, primary sources, waste materials. The primary sources consulted included the Scopus database, as well as publisher-specific platforms such as the American Chemical Society, IOP Science, MDPI, the Royal Society of Chemistry, Taylor & Francis Online, ScienceDirect, SpringerLink, and Wiley Online Library. Additionally, the search covered freely accessible online papers, databases, and institutional reports to obtain detailed information. Journal articles and books were selected based on full-text evaluations. Comprehensive coverage of the relevant literature was ensured through cross-referencing citations and examining any supplementary materials provided. Particular attention was paid to methodological approaches, key findings, and the relevance of the data to the current research context, providing the main foundation for subsequent analysis and discussion of ruthenium sources and recovery methods.

## 3. Primary Ruthenium Resources and Production

### 3.1. Occurrence

Ruthenium is one of the rarest elements on Earth, although it is not the rarest of the platinum group metals. Its abundance in the bulk continental crust is estimated at 0.6 ppb [15], while the remaining platinum group metals are present at 0.14 ppb Rh [16], 1.5 ppb Pd, 0.04 ppb Os, 0.04 ppb Ir, and 1.5 ppb Pt [15]. In nature, ruthenium rarely occurs in a pure form [17]; instead, it is typically found in association with other platinum group metals [18,19,20,21,22,23,24,25,26,27,28,29,30,31]. It occurs (Table 1) mainly as microscopic inclusions in base-metal sulfide deposits (e.g., pentlandite (Fe,Ni)_9_S_8_, pyrrhotite Fe_1−x_S, and chalcopyrite CuFeS_2_) and in chromite FeCr_2_O_4_-bearing rocks (chromitite), either as minerals (laurite (Ru,Os)S_2_, ruarsite RuAsS) [17] or as component of natural Os–Ir–Ru alloys (up to 10–20% Ru) [31].

Anomalously high concentrations of ruthenium have been reported in natural nuclear reactors (associated with uranium deposits) discovered in the 20th century in Gabon, Africa [32]. As a product of nuclear fission, ruthenium accumulates in these systems, and analyses of samples from the Oklo and Bangombé reactor zones indicated that its total concentrations can range from about 1.2 ppm in black shales to values as high as 830 ppm in uraninite.

A noteworthy aspect is the extraterrestrial occurrence of ruthenium. For example, concentrations of 690 ppb have been reported in CI chondrites [33], 2–25 ppm in iron meteoriites [34], while chromite from Martian meteorites can contain as much as 100–120 ppm [35]. Ruthenium has also been identified in the solar spectrum and in some stellar spectra, with its abundance in the Solar System estimated at 1.8–1.9 atoms per 10^6^ atoms of silicon [34].

### 3.2. Primary Production

**Figure 3:**
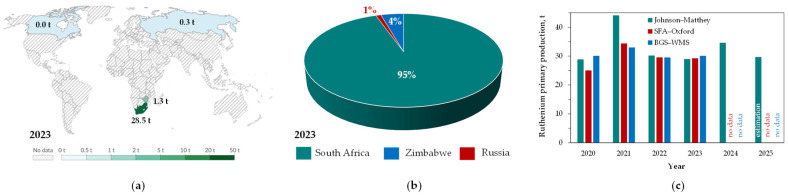
Ruthenium primary production: (**a**) producing countries and production volumes in 2023 (adapted from [36] under License CC BY 4.0), (**b**) production share by country in 2023 (based on [35]), (**c**) total production from 2020 to 2025 (based on data from Johnson-Matthey [1], SFA–Oxford [37], BGS—World Mineral Statistics [36]).

Ruthenium is obtained principally as a by-product during the recovery of other platinum group metals. Due to the concentration of ruthenium-bearing deposits in certain regions (Figure 3a,b), the economic extraction of this element is predominantly confined to South Africa (Bushveld Complex), with significantly lower production levels in Zimbabwe (Great Dyke) and Russia (Norilsk-Talnakh). Canada, once a traditional supplier, ceased production a few years ago [1,36]. Nonetheless, global annual ruthenium production from primary sources remains relatively stable at about 30 tons, although specific values may vary depending on the data source (Figure 3c).

The future availability of ruthenium is difficult to determine, as world resources are hard to assess due to the lack of detailed data, with the latest estimations suggesting a total of near 4500 tons [30]. Indeed, this is more complex mechanism by its reliance on the extraction of other elements, such as platinum or nickel.

### 3.3. Technologies

Ruthenium can be sourced from three types of ores [38]: (i) ores primarily mined for PGMs, with other metals (Ni, Cu, Co) as by-products of relatively low economic value (e.g., South Africa, Zimbabwe); (ii) Ni-Cu sulfide ores, the dominant source of PGMs, where they are recovered as by-products (e.g., Russia, Canada); and (iii) miscellaneous ores, which are not currently mined and are considered accessory. The extraction technologies of precious metals from these sources vary in their processing specifics and flowcharts [38,39,40,41], with their details remaining guarded.

During large-scale processing of ruthenium-bearing Ni-Cu sulfide ores, the initial stages lead to the concentration of PGMs, employing a wide range of process strategies [38]. One of the pathways [40] involves crushing the ore (5–8 ppm PGM), followed by flotation to achieve a flotation concentrate (10–15 ppm PGM). Further smelting (150 ppm PGM) and magnetic separation result in the accumulation of PGMs in the magnetic Ni-Cu-Fe metallic phase. After the dissolution of base metals (Cu, Ni, Fe), a final PGM concentrate (60% PGM) is obtained. Such treatment was employed by Falconbridge (Canada) in its Chlorine Process [38] or at the Rustenburg Mines Plant [39] as examples.

Another source of PGM concentrates can be anodic slimes from electrorefining, which represent the final stage of nickel and copper production from sulfide ores. For example, at the Norilsk metallurgical company (Russia), these slimes are roasted and leached separately to recover nickel and copper, respectively [38]. The leach residues are then combined and smelted to produce anodes for subsequent electrorefining. At this stage, three types of PGM concentrates are generated: (i) 65% (Pt + Pd) from anode slime, (ii) 2.5–3.5% (Ru + Rh) from copper sponge, and (iii) 10–20% Ir from spent electrolyte.

**Figure 4:**
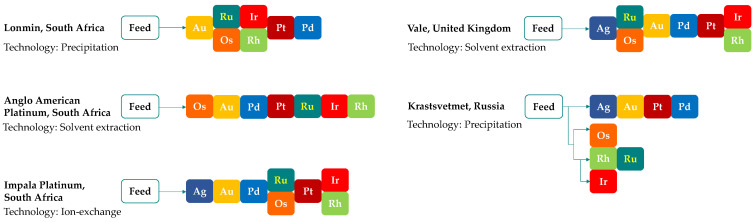
Sequence of ruthenium separation (row elements sequential, vertical together) from PGM concentrates leached in HCl + Cl_2_, at different refineries (based on [41]).

**Figure 5:**
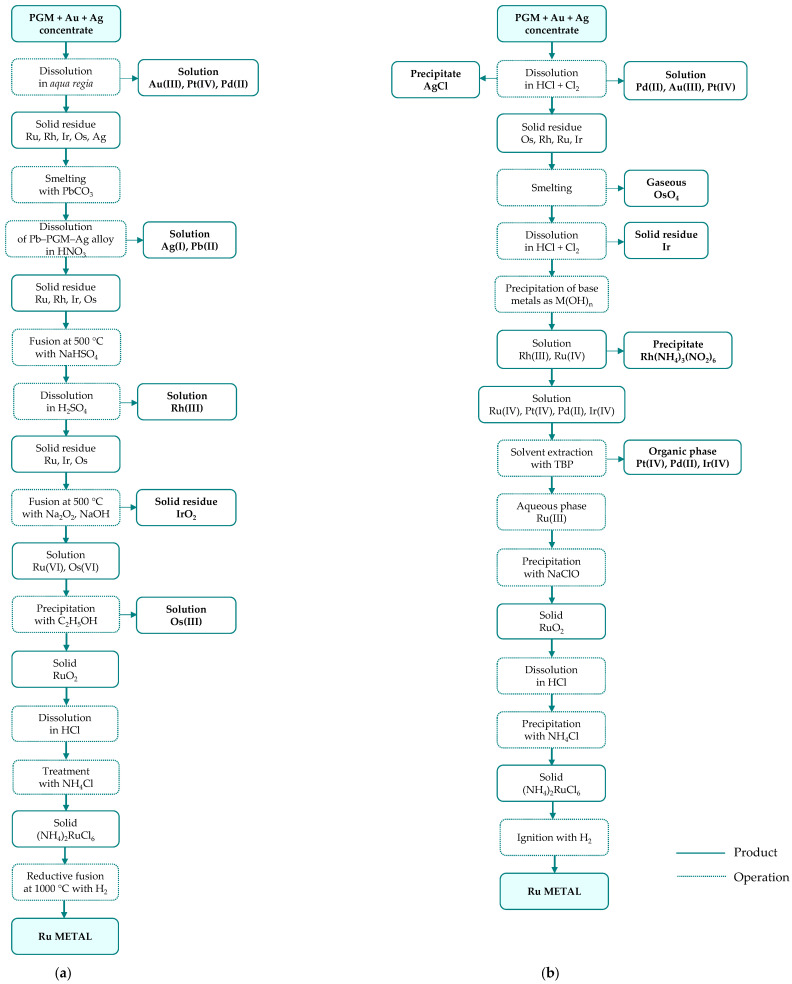
Ruthenium separation from platinum group metal concentrate through precipitation technology developed by: (**a**) Inco Acton Refinery; primary raw material: Ni-Cu sulfide ore (based on [38]); (**b**) Krastsvetmet Refinery; raw material: anodic slimes from nickel and copper electrorefining at Norilsk (based on [41]).

**Figure 6:**
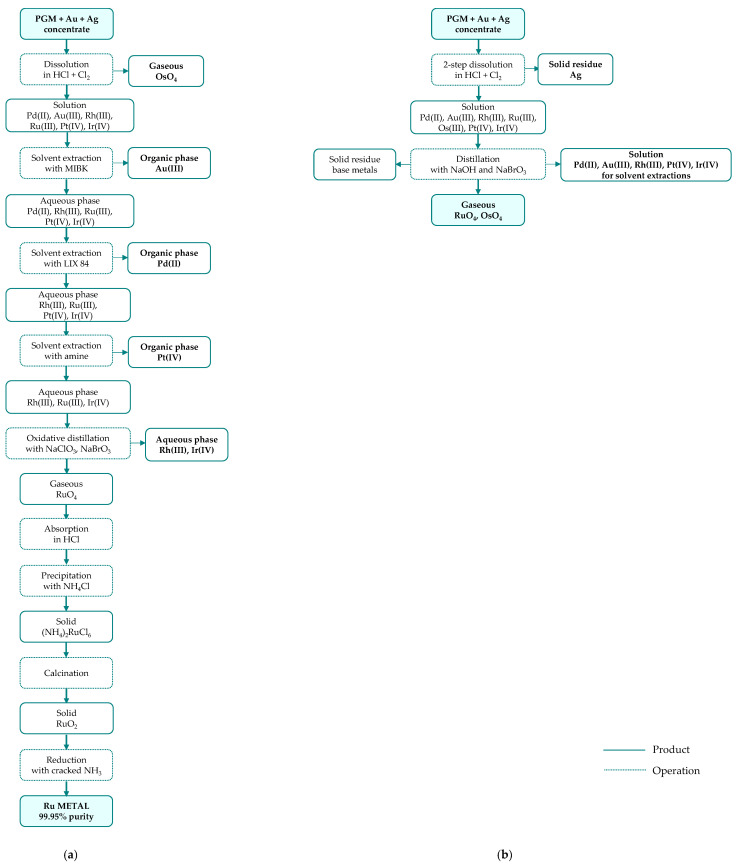
Ruthenium separation from platinum group metal concentrate through solvent extraction technology developed by: (**a**) Matthey Rustenburg Refiners and Anglo American Platinum; primary raw material: Ni-Cu sulfide ore (based on [40,41]); (**b**) Mintek ConRoast Process; primary raw material: high chromite ore (based on [38]).

**Figure 7:**
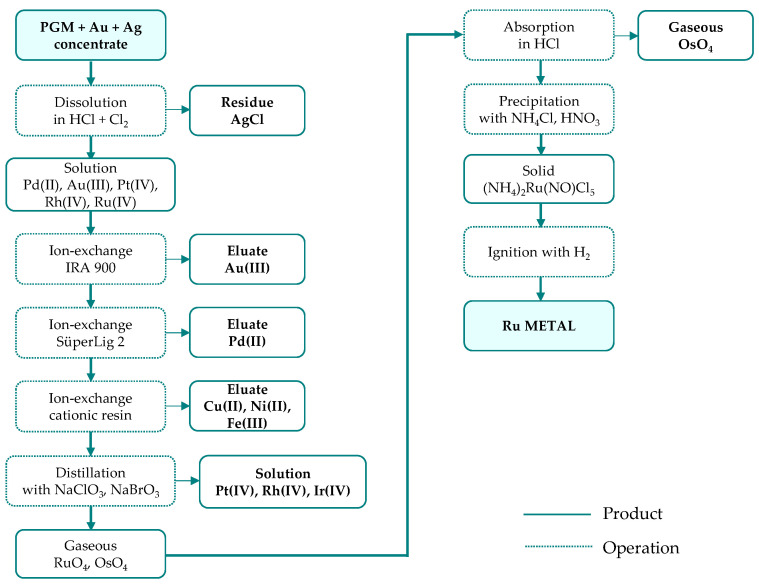
Ruthenium separation from platinum group metal concentrate through ion-exchange technology developed by Impala Platinum; primary raw material: converter matte (based on [41]).

The PGM concentrates contain 15–75% of precious metals (PGM, Au, Ag) and serve as the feedstock for ruthenium production through leaching, followed by multi-stage separation and refining. Although refining routes differ in detail, they are all based on the same general principle, involving primary separation, secondary purification, and final reduction to the metallic form. Most commonly, hydrochloric acid (4–6 M) with chlorine gas as oxidant is used for concentrate dissolution; however, the exact leaching conditions are process-specific and typically range from 65 to 98 °C at atmospheric pressure to 75–120 °C under elevated pressure (up to 3–4 bar) [41]. The separation of precious metals is carried out in different sequences (Figure 4), depending on the applied technique (precipitation—Figure 5, solvent extraction—Figure 6, or ion exchange—Figure 7) used to separate the two most abundant metals (platinum and palladium) and thus on the reagents employed.

In many refineries, ruthenium recovery from the leachate is carried out as part of a stepwise metal separation process, in which individual metals are removed from the solution one after another in the main separation line, followed by a purification stage [41]. A slightly different approach is applied at Russian Krastsvetmet refinery, where ruthenium is separated alongside rhodium and iridium in a circuit distinct from the platinum and palladium circuit (Figure 5b). In this process, feed material with low platinum and palladium contents is smelted, and the resulting alloy (mainly osmiridium), is dissolved in a highly concentrated acid–chlorine system, with simultaneous removal of volatile osmium oxide. The obtained solution is first processed to recover rhodium, and then purified from other PGM and finally directed to the ruthenium recovery stage.

Ruthenium dissolved from concentrate can exist in acid chloride system as complex ion RuCl_6_^2−^:

Ru + 2HCl + 2Cl_2_ → H_2_RuCl_6_ (1)

Its separation is carried out through reactive distillation at 80–90 °C [41], along with osmium, due to the formation of highly volatile oxides, RuO_4_ and OsO_4_ (vapor pressure of about 10 mmHg at 25 °C [9]). The extraction process uses sodium chlorate NaClO_3_ and then sodium bromate NaBrO_3_ as oxidizers:

6RuCl_6_^3−^ + 5XO_3_^−^ + 9H_2_O → 6RuO_4_↑ + 5X^−^ + 36Cl^−^ + 18H^+^ (2)

2RuCl_6_^3−^ + 2XO_3_^−^ + 2H_2_O → 2RuO_4_↑ + X_2_↑ + 12Cl^−^ + 4H^+^ (3)

where X represents chlorine or bromine.

Both oxides (ruthenium and osmium) are carried away by air passed through the solution to a scrubbing column, where diluted HCl traps and reduces the oxides to non-volatile aqueous forms:

RuO_4_ + 10HCl → H_2_RuCl_6_ + 4H_2_O + 2Cl_2_↑ (4)

The process continues until the ruthenium concentration is reduced to less than 10–250 mg/L [41].

The metals are then separated by reoxidizing osmium to gaseous OsO_4_, while ruthenium remains in solution as RuCl_6_^2−^. It is subsequently precipitated with ammonium chloride to form ammonium hexachlororuthenate(IV) (NH_4_)_2_RuCl_6_, which is then reduced to pure ruthenium metal.

H_2_RuCl_6_ + 2NH_4_Cl → (NH_4_)_2_RuCl_6_↓+ 2HCl (5)

(NH_4_)_2_RuCl_6_ + 2H_2_ → Ru + 2NH_3_↑ + 6HCl↑ (6)

Alternatively, ruthenium from the HCl solution can be precipitated with NH_4_Cl and HNO_3_ to form ammonium pentachloronitrosylruthenate(II) (NH_4_)_2_Ru(NO)Cl_5_:

H_2_RuCl_6_ + 2NH_4_Cl + HNO_3_ + HCl→ (NH_4_)_2_Ru(NO)Cl_5_↓ + 2Cl_2_ + 2H_2_O (7)

The salt is then ignited to produce ruthenium sponge (a method used in the Impala Platinum’s ion-exchange technology).

Typical recoveries of high-purity ruthenium in the refinery technologies are in the range of 90–99.5% [41].

Distillation in glass-lined vessels is a common method in ruthenium production, but it poses risks due to the formation of explosive nature of chlorine oxide and release a highly toxic ruthenium and osmium tetroxides [9]. Therefore the equipment is designed to protect workers from such accidents, with strict monitoring and control measures in place [41].

Although refineries employ practically the same ruthenium recovery route, it should be noted that the application of solvent extraction or ion-exchange methods for the recovery of other noble metals significantly increases separation selectivity, maintains higher process kinetics, and provides substantially higher first-pass yields, while facilitating process automation compared with precipitation. However, both approaches require the use of more dilute solutions, which results in higher solution volumes and a greater number of evaporation stages than in precipitation-based processing.

## 4. Ruthenium Recovery from End-of-Life Catalysts

### 4.1. Spent Membrane Electrode Assemblies

The membrane electrode assembly MEA is a key component of proton exchange membrane fuel cells PEMFC and alkaline water electrolysers AWE [42,43]. It is a multilayer sandwich structure in which the anode and cathode catalyst layers (PGM particles dispersed on carbon support) are separated by a polymer electrolyte membrane (e.g., Nafion) acting as the ion-conducting medium. In these assemblies, ruthenium and ruthenium-based materials (alloys, oxides) are commonly used as anode catalysts owing to their high activity toward the oxygen evolution reaction (about 0.01–0.03 mg Ru/cm^2^ [43]), or as alloying component in platinum-based catalysts to improve catalytic performance for hydrogen evolution. The main component of the PGM catalyst particles is typically platinum (0.25–0.45 mg/cm^2^ [44]) with its mass ratio of 0.14% in PEMFC [45]. The contribution of ruthenium to the overall material composition is ten times lower, at about 0.01% in PEMFC and 0.02% in AWE [45]. This difference in the relative contents of both metals leads to spent PEMFCs and AWEs being recycled predominantly for platinum recovery [46,47,48] as catalyst material accounts for 40–60% of the total costs of the PEMFC stacks [49,50]. The overall strategy for ruthenium recovery from spent fuel cells or water electrolysers [48] involves manual separation of the membrane electrode assembly, followed by its delamination to detach the cathodic and anodic catalytic layers. The ruthenium-rich material (primarily the anode catalyst) can then be treated using conventional hydrometallurgical or pyrometallurgical technologies within a closed-loop recycling scheme, or subjected to one of the newly developed techniques.

**Figure 8:**
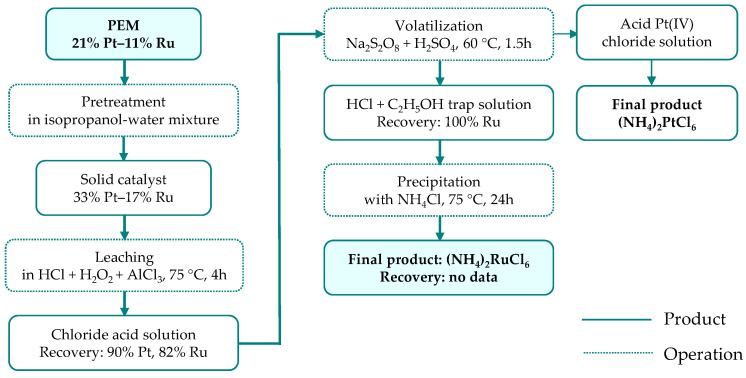
Ruthenium separation from PEM membrane through leaching-volatilization-precipitation method (based on [52]).

Sandig-Predzymirska et al. [51,52] proposed a multistep approach for the recovery of platinum and ruthenium from proton exchange membranes containing PtRu alloy catalyst particles (Figure 8). The process began with a pretreatment in an alcohol–water mixture, which enabled the separation of PtRu alloy nanoparticles from the carbon-based membrane support. The recovered catalyst was subsequently subjected to leaching in hydrochloric acid, with hydrogen peroxide acting as the oxidizing agent and aluminum chloride serving as a cost-effective additive [51] that partially reduced the need for highly concentrated HCl. Ruthenium was then selectively separated from platinum via a volatilization step to convert RuCl_6_^2−^ species into gaseous RuO_4_ [52]. The efficiency of this transformation strongly depended on the oxidizing agent employed and followed the order: HNO_3_ (no Ru recovery) < (NH_4_)_2_S_2_O_8_ (~15% Ru recovery, 2 h) < K_2_S_2_O_8_ (100% Ru recovery; 2 h) < Na_2_S_2_O_8_ (100% Ru recovery, 1.5 h). The volatile RuO_4_ was captured in an HCl solution containing ethanol as a reducing agent, leading to the formation of RuCl_6_^2−^ and/or RuCl_6_^3−^ complex ions. Alternative trapping media (methanesulfonic and oxalic acids) were also examined but proved less effective than the acid–alcohol system. In the final recovery stage, ruthenium salt was precipitated using NH_4_Cl, but the overall recovery efficiency of the process was not reported.

Notably, the isolated products, (NH_4_)_2_RuCl_6_ and (NH_4_)_2_PtCl_6_, were subsequently employed as precursors for catalyst resynthesis [52]. By combining both salts with a carbon suspension and treating the mixture under elevated temperature and pressure, PtRu nanoparticles were obtained. Although the resulting material did not reach the targeted alloy composition, the study clearly demonstrates the potential of recovered platinum-group metal salts for the preparation of electrocatalysts suitable for further electrode fabrication.

**Figure 9:**
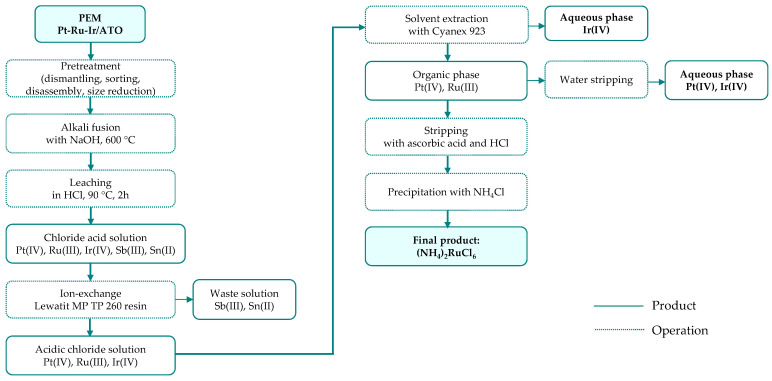
Ruthenium separation from PEM membrane through solvent extraction route (based on [53]).

Otgonbayar et al. [53] investigated the recovery of metals from two types of PEM electrocatalysts, namely PtRu and IrRu/ATO (where ATO is antimony-doped tin oxide support). The process was first optimized with synthetic solutions and subsequently validated using real PEM leachates. To enhance catalyst leachability, the initial material was subjected to alkaline fusion using NaOH or a NaOH/NaNO_3_ mixture, followed by acid leaching in HCl (6M, 90 °C, 2 h). Subsequent solvent extraction with Cyanex 923 (dissolved in diesel) resulted in complete or partial extraction of platinum (90–100%), ruthenium (80–97%), and iridium (46%), accompanied by the co-extraction of tin and antimony. To suppress the latter, an ion-exchange step employing Lewatit MP TP 260 resin was introduced prior to solvent extraction. After the separation of iridium from the platinum–ruthenium stream, ruthenium was further isolated using ascorbic acid and HCl, ultimately yielding (NH_4_)_2_RuCl_6_ as the final product. Although the individual steps of the solvent extraction process were investigated in detail, the overall ruthenium recovery efficiency for the proposed flowsheet (Figure 9) was not reported.

**Figure 10:**
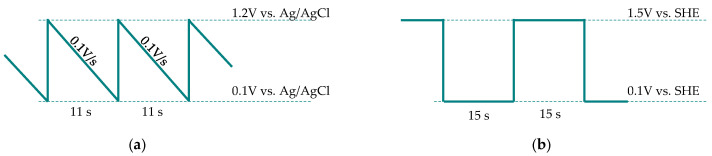
Optimal electrochemical modes for ruthenium dissolution from PEM membrane through potentiodynamic electrolysis in 1 M HCl: (**a**) saw-tooth potential wave (based on [54]); (**b**) square potential wave (based on [55]).

Alternatively, electrochemical pathways were also developed [54,55,56]. Shiroishi et al. [54] demonstrated that a saw-tooth potential waveform dissolved platinum from membrane electrode assemblies in 1 M HCl about fifteen times faster than either constant-potential operation or chemical treatment using 1 M HCl with H_2_O_2_. The same approach was subsequently applied to PtRu catalysts, resulting in the dissolution of nearly 80% of ruthenium within 600 s at room temperature (Figure 10a).

In turn, Kanamura and Yagyu [55] investigated a square-wave potential regime consisting of alternating oxidation and reduction steps (Figure 10b). Under these conditions, metal dissolution occurred during the oxidation stage, accompanied by gradual formation of passivating oxide layer, whereas during the reduction stage the oxide was converted back to the metallic phase. The efficiency of ruthenium dissolution depended strongly on the applied potentials, the cycle duration and the HCl concentration. Under optimal conditions (Figure 10b), 98% of ruthenium was dissolved within 50–60 min of electrolysis in 1 M acid at room temperature, whereas only about 5% dissolution was achieved under potentiostatic conditions. Notably, ruthenium exhibited slightly higher dissolution efficiencies than platinum, which was attributed to the formation of volatile RuO_4_ species.

Patel et al. [56] examined the electrochemical dissolution of ruthenium in acidified halide media (4 M NaCl, NaBr, and NaI) using various voltammetric techniques. Although dissolution was achievable at room temperature, the rates were insufficient to render the process attractive for large-scale applications. Among the investigated electrolytes, only the acidified chloride medium enabled efficient ruthenium dissolution at potentials below the onset of oxygen evolution, whereas higher anodic potentials were required in the bromide medium. In both cases, parasitic side reactions related to halogen gas evolution were observed. By contrast, the iodide-based medium did not facilitate ruthenium dissolution. In all cases, the process was further impeded by the formation of a passivating oxide layer on the metal surface.

It should be noted that electrochemical oxidation of spent catalysts provides not only a significantly higher dissolution rate compared with conventional chemical leaching in acidic chloride solutions, but it eliminates the need for high hydrochloric acid concentrations and pretreatment stages.

### 4.2. Spent Ceramic Supported Catalysts

Ruthenium-based catalysts are widely used for large-scale hydrogenation, amine formation, oxidation and Fischer–Tropsch reactions [57,58]. The catalytic particles are typically dispersed within porous oxide supports (zirconia, alumina, silica, carbon, etc.) and can contain 0.1–30% ruthenium. This makes them highly attractive secondary resources for ruthenium recovery, although such materials require often combined hydro- and pyrometallurgical treatment.

**Figure 11:**
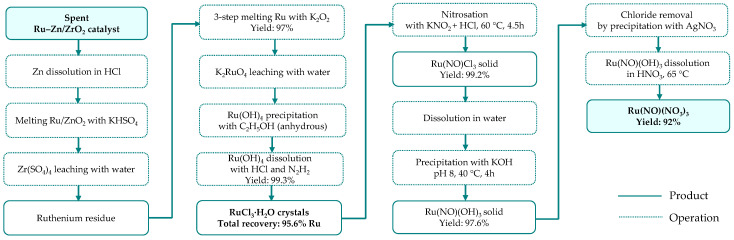
Ruthenium recovery as ruthenium nitrosyl nitrate from spent Ru–Zn/ZrO_2_ catalyst (based on [61]).

Ru–Zn/ZrO_2_ catalysts are used in the industrial selective hydrogenation of benzene to cyclohexene [59]. These catalysts are characterized by a simple composition and relatively high ruthenium loadings, typically in the range of 5–15% [57,58,59,60,61,62]. Liu et al. [61] developed a multistage method for the synthesis of ruthenium nitrosyl nitrate Ru(NO)(NO_3_)_3_ from a spent catalyst (11% Ru, 5.8% Zn, 61% Zr). The process was divided into two main phases: (i) the preparation of purified ruthenium chloride from the waste catalyst and (ii) the conversion of the chloride intermediate into the final high-purity product. The effects of temperature, reagent concentration, reaction time, and pH on the yield and purity of intermediates and the final product were systematically examined, allowing the identification of optimal process conditions (Figure 11). Under these conditions, the total yield of Ru(NO)(NO_3_)_3_ reached 92%, while the ruthenium content in the high-purity product was 32.2%.

Zhang et al. [62] proposed a relatively simple procedure for ruthenium recovery. The process began with the selective leaching of zinc using H_2_SO_4_, achieving 88% Zn extraction while only 0.06% of ruthenium was dissolved under optimal conditions (1 M acid, 60 °C, 1 h, L/S 4). This was followed by roasting the solid residue with NaHSO_4_·H_2_O (350 °C, 40 min) and subsequent water leaching of Zr(SO_4_)_2_, which removed 96% of zirconium. The remaining residue, enriched in ruthenium (79.3% Ru), was then subjected to alkaline fusion with NaNO_3_ and NaOH (2:1, 400 °C, 2 h), converting ruthenium into Na_2_RuO_4_. The resulting product was distilled (90 °C) in the presence of a NaClO_3_–H_2_SO_4_ mixture, oxidizing ruthenium from Na_2_RuO_4_ to volatile RuO_4_. The ruthenium tetroxide was absorbed using a conventional trapping method (18% HCl and 0.5% C_2_H_5_OH solution), achieving a distillation efficiency of 97% and yielding RuCl_3_·H_2_O as the final product.

**Figure 12:**
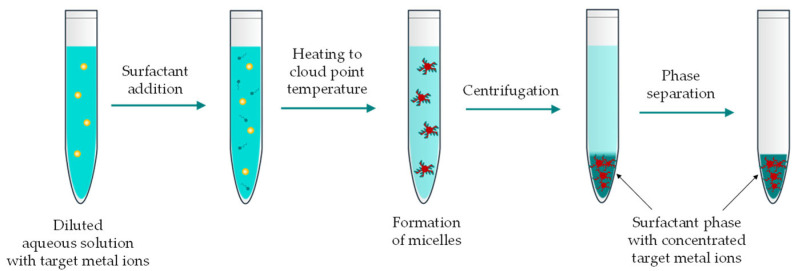
Scheme of cloud point extraction.

Suoranta et al. [63] adapted a microwave-assisted leaching and cloud point extraction method for ruthenium recovery from a Ru/Al_2_O_3_ catalyst (0.5% Ru). The process involved leaching with HCl, HNO_3_, or aqua regia (L/S ratio of 40), achieving 90–95% ruthenium recovery at temperatures of 90–210 °C (15 min heating, 10 min at the target temperature, followed by natural cooling to room temperature). Under optimal leaching conditions (120 °C, HCl leachant), 95% ruthenium recovery was achieved, with only 30% of aluminum dissolved. The leachate was then diluted to a 1 M HCl concentration and subjected to cloud point extraction, a method that uses small amounts of relatively environmentally friendly reagents to form neutral complexes with metal ions. During this process, hydrophobic metal complexes are extracted into micelles formed by the added surfactant, and the surfactant-rich phase is separated from the aqueous phase by heating above the cloud point temperature, which simultaneously concentrates the target metal ion (Figure 12).

Cloud point extraction of ruthenium ions was carried out with a non-ionic surfactant Triton X-100 and 2-mercaptobenzothiazole 2-MBT as chelating agent. After several minutes, SnCl_2_–HCl solution was added to accelerate the exchange of chloride ions of ruthenium chlorocomplexes with 2-MBT. Following heating and cooling, the aqueous phase was decanted, leaving a ruthenium-rich phase (~1 mL) at the bottom of the tube. This approach resulted in a ruthenium recovery of 66 ± 11% from leachates containing 9–40 mg Ru^3+^/L, with only 4% aluminum extraction at its concentrations of 330–1200 mg Al^3+^/L in the solution. However, despite achieving some degree of preferential ruthenium recovery, its selectivity appears insufficient. Additionally, no data were provided on the final isolation of ruthenium from the surfactant phase.

On the other hand, Zhang et al. [64] used a similar method for ruthenium recovery from an HCl solution, employing a 2-mercaptobenzothiazole-functionalized ionic liquid [C_6_mim][2MBT] as the extractant. The ruthenium(III) ions were extracted into a surfactant phase of octylphenoxypolyethoxyethanol TX-114, achieving 82% recovery under optimal conditions. This study utilized a standard ruthenium(III) salt solution, without the presence of other contaminating metal ions. However, the paper demonstrated a method for ruthenium stripping from the surfactant-rich phase by precipitating metallic particles using a sodium borohydride NaBH_4_ solution.

## 5. Ruthenium Recovery from Metallurgical By-Products and Waste

### 5.1. Nickel and Copper By-Products

Although the recovery of ruthenium from nickel-copper sulfides is well developed and implemented on an industrial scale, advancements continue to emerge regarding PGM recovery at different stages of hydrometallurgical operations. van Schalkwyk et al. [65] analyzed the behavior of ruthenium (Rh and Ir) during the leaching of Ni-Cu-Fe-S converter matte with varying iron concentrations (0.53–5.72%) under both oxygenated and non-oxygenated conditions. These studies were initiated by observations that iron content in the matte has a significant impact on the performance of the first stage atmospheric leach, in which one of the primary goals is to precipitate PGEs from spent copper electrowinning electrolyte (acid nickel–copper sulfate solution). It was found that complete ruthenium precipitation (as well as iridium and rhodium) occurred only under oxygenated conditions for low-iron matte, whereas only 38–49% of ruthenium could be transferred to the PGM concentrate when high-iron material or non-oxygenated leaching conditions were tested. Additional studies [66] showed that the precipitation and leaching kinetics of these PGMs are primarily influenced by temperature (116 or 130 °C), but less so by pressure (7 or 9 bar) and sulfuric acid concentration (140 or 165 g/L). Notably, such behavior is crucial for the production of PGM concentrates for further processing.

Further studies [67] evaluated precipitation behavior during the formation of an iron sludge via hydrolysis from synthetic nickel sulfate leach solutions (10–14 mg Ru^3+^/L), produced in the first leaching stage of matte in base metal refineries. It was found that average ruthenium precipitation via hydrolytic reactions reached 98.1% at pH 4. The iron valence state and total metal concentration as well as seeding had no significant effect on the metal (Ru, Rh, Ir) precipitation from the leach solution. In turn, Mulwanda and Dorfling [68] analyzed the precipitation of Ru (and Rh, Ir) from leach solutions produced in the final leaching stage of Ni-Cu matte leaching. The pregnant solution contained approximately 220 mg/L Ru^3+^ (and 39 ± 4 mg/L Ir or Rh), but a maximum of 87% of ruthenium could be precipitated using thiourea (200% excess, 80 °C, 7 bar, 4 h). Although the recovery was not complete, it was improved compared to the use of sulfurous acid traditionally employed in Se/Te removal stages.

Nagai et al. [69,70] developed a method for ruthenium recovery from copper anodic slime. During slime treatment with sulfuric acid, ruthenium concentrates in the Se/Te residues (2% Ru). Both chalcogenides were dissolved in NaOH solution (12% Ru in solid residue), while copper was dissolved with sulfuric acid. The solid residues, enriched in Ru (20%) and Rh, were then chlorinated (to remove Se and Te) and roasted with NaCl to convert them into water-soluble chlorides (48% Ru). This chloride solution served as the starting material for traditional oxidizing distillation, followed by precipitation of (NH_4_)_4_[Ru_2_Cl_10_O] and pyrolysis to obtain ruthenium powder. Unfortunately, although the composition of the individual intermediates was reported in detail, no data on recovery values were provided.

Mwase et al. [71] investigated combined bioleaching and cyanidation of low-grade concentrate originating from a typical PGM concentrator plant. The material contained 4.4 ppm Ru and 2.6 ppm Rh, along with higher concentrations of Pt (12 ppm) and Pd (8.2 ppm). The slurry was initially treated with a mixed culture of thermophilic and mesophilic bacteria (predominantly *Sulfolobus metallicus* and *Ferroplasma cupricumulans*) at 65 °C to remove copper (52%), nickel (95%), and cobalt (85%). At this stage, 19% of ruthenium and 38% of rhodium were also co-extracted. The residuals were then further leached in a column with sodium cyanide (NaCN, 0.15 M, sodium carbonate buffer of pH 10, 23 °C, 21 days). However, during this stage, only 3.4% of ruthenium was released into the solution, while the process was efficient for extracting the remaining PGMs. This difference between the cyanidation behavior of ruthenium and other noble metals originated from the high chemical inertness of ruthenium, resulting in very slow complex formation under leaching conditions leading to low dissolution rates.

### 5.2. Red Mud

Red mud is a solid waste generated during the production of aluminum oxide using the Bayer method [72]. It is estimated that each ton of alumina produced generates 0.4 to 2 tons of waste, totaling approximately 300 million tons per year globally. Red mud is primarily composed of 4–55% Fe_2_O_3_, 6–27% Al_2_O_3_, 3–24% SiO_2_, 2–17% TiO_2_, up to 40% CaO, and up to 10% Na_2_O [73]. Although this material is hazardous and is typically disposed of in industrial practices, it can also represent a valuable source of rare earth elements (500–1700 ppm), scandium (20–160 ppm), gallium (30–570 ppm). Interestingly, Ma et al. [74] recently identified 0.1% ruthenium in red mud from an alumina factory in China. They then optimized the leaching conditions for this metal, using inorganic acids as leaching agents. The efficiency of ruthenium recovery increased in the following order: HNO_3_ (34%) < H_2_SO_4_ (59%) < HCl (83%) < H_3_PO_4_ (87%). Further investigation of process variables such as H_3_PO_4_ concentration (3–7 M), temperature (20–90 °C), L/S ratios (6–16), time (1–8 h), and stirring rate (300–700 rpm) revealed the optimal operating parameters (5 M acid, 90 °C, 6 h, L/S 12, 500 rpm), under which 93.5% of ruthenium could be dissolved from the waste. The obtained solution contained 0.1 g/L ruthenium and required further separation due to the high concentration of base metals such as Fe (39 g/L), Al (7 g/L), and Ca (8 g/L).

## 6. Ruthenium Recovery from Diluted Solutions

### 6.1. Wastewaters

Ruthenium-containing wastewaters are generated in various industrial sectors, including (i) integrated circuit manufacturing [75], where chemical–mechanical planarization of ruthenium barrier layers is employed to remove excess material [76]; (ii) electroplating processes in the semiconductor industry or for decorative applications [7,77,78,79,80,81]; and (iii) acetic acid production using ruthenium complex Ru(CO)_4_I_2_ as promoter (Cativa process) [81,82,83,84]. Such acidic or alkaline effluents typically contain low concentrations of ruthenium species (0.2–3 g/L), along with other metallic contaminants (e.g., iron, copper, zinc, nickel [79,80]), different inorganic ions (nitrate [79], chloride [78], sulfate [77]) or organic compounds. Given that ruthenium is both a valuable and environmentally hazardous element, dedicated recovery methods have been developed that are specifically tailored to dilute metal ion solutions (Table 2).

Ruthenium, as a noble metal with a standard potential of the Ru/Ru^3+^ redox couple of 0.7 V, is amenable to recovery by cementation using more electrochemically active metals such as aluminum (−1.6 V for Al/Al^3+^) or zinc (−0.7 V for Zn/Zn^2+^). Le et al. [75] demonstrated that ruthenium could be almost completely recovered from alkaline wastewater (pH 8.7) originating from integrated circuit manufacturing by cementation with aluminum powder. However, the final product consisted of metallic ruthenium particles coagulated within an Al(OH)_3_ precipitate. The process efficiency and rate were found to depend primarily on aluminum particle size (53–177 μm), the Al-to-Ru molar ratio (0.5–2), and temperature (25–65 °C), while being less sensitive to the initial ruthenium concentration (20–200 mg/L). Nevertheless, at least a twofold excess of the cementing metal relative to the stoichiometric requirement was necessary. In contrast, Aktas et al. [77] investigated ruthenium cementation from acidic sulfate solutions using zinc powder and found that a minimum tenfold excess over the theoretical zinc demand was required to achieve near-complete recovery. This high zinc consumption was attributed to parallel zinc dissolution by free sulfuric acid, whereas the addition of NaCl acted as a process accelerator, enabling comparable ruthenium recovery with more than a twofold reduction in zinc consumption.

Recovery processes from highly diluted solutions commonly rely on adsorption onto porous materials. Yue et al. [78] synthesized an amorphous magnesium–calcium carbonate phosphate Mg-CCP adsorbent exhibiting ultrafast ruthenium uptake kinetics (30 s). This low-cost material, characterized by a spherical nanoshell morphology and a high specific surface area (50.2 m^2^/g), enabled efficient ruthenium recovery even from solutions containing as high as 3 g/L Ru. Subsequent calcination converted the Ru-loaded adsorbent into oxide phases, which, after washing with diluted HCl, yielded RuO_2_ (95% purity) as the final product. In a different approach [79], a silica–polymer-based adsorbent modified with TRPO demonstrated high selectivity toward ruthenium adsorption from nitrate solutions (0.1 M HNO_3_). Under optimized conditions and in the presence of a NaNO_2_ additive (0.05 M), no adsorption of Zn, Cu, or Ni ions was observed, while Fe uptake was limited to 1.4%. The material exhibited a high adsorption capacity for ruthenium of approximately 55 mg/g.

Alternative adsorption routes exploit the metal-binding capacity of bacterial biomass. Colica et al. [80] investigated ruthenium biosorption using *Rhodopseudomonas palustris* from both acidic and alkaline real wastewaters. Ruthenium uptake was selective with respect to competing metal cations (Cu, Ni, Zn) and was slightly higher in alkaline solutions. Interestingly, pronounced differences were observed at the metal recovery stage from the loaded biomass, depending on the post-treatment method (centrifugation, lyophilization, mineralization), resulting in final ruthenium recoveries of 72% and 42% for acidic and alkaline solutions, respectively. Significantly higher sorption performance was reported for exopolymers produced by *Pseudomonas aeruginosa*, which were capable of adsorbing 5–10 times more ruthenium, achieving sorption capacities close to 200 mg/g from acidic chloride solutions [81]. Other studies developed fiber-type adsorbents modified with chitosan and various bacterial biomasses, including *Corynebacterium glutamicum* [82,83] and *Escherichia coli* [84], for ruthenium recovery from acetic acid wastewaters. However, metal recovery from these biosorbents required complex downstream processing, involving incineration at elevated temperature (800 °C) followed by treatment with aggressive reagents (NaClO, KMnO_4_, aqua regia) [83], which raises concerns regarding the practical applicability of such processes. Although biosorbents require relatively long adsorption times to reach equilibrium (often exceeding several hours), they exhibit adsorption capacities that are approximately one order of magnitude higher than those of ion-exchange resins [82,84]. In contrast, ion-exchange resins typically achieve maximum ruthenium uptake within shorter contact times (a few hours), but with adsorption capacities that are several to tens of times lower.

### 6.2. Solvent Extraction

Solvent extraction is an effective and selective method for separating PGM ions from multicomponent solutions, with simultaneous preconcentration occurring during the stripping step. This feature is essential for the industrial application of this technique in refineries (Figure 6). However, most studies focus on the solvent extraction of primary PGMs such as platinum and palladium [85], while less attention is given to ruthenium, as it is often separated via distillation. Nevertheless, alternative solvent extractants from different media (Table 3) have also been tested, employing both traditional organic extractants [86] and ionic liquids [87].

A typical aqueous medium for solvent extraction is acid chloride solutions. Panigrahi et al. [88] compared the extractability of RuCl_4_^−^ using two anionic extractants: a tertiary amine Aliquat 336 and a quaternary amine Alamine 336. They observed that the addition of NaCl (0.5–4.5 M) significantly increased ruthenium extraction with Aliquat 336 (from 50% to 95%), while only slightly improving extraction with Alamine 336 (55–70%). Further analysis of extraction with Aliquat 336 revealed that, although the extraction step was highly efficient, stripping the ruthenium species was challenging. Among the 11 stripping agents tested (water, inorganic and organic acids, sodium hydroxide, salts), only sodium carbonate Na_2_CO_3_ showed high efficiency, although the stripping efficiency was limited to 77–84%.

Song et al. [89] employed Aliquat 336 as an extractant, dissolved in the ionic liquid tri-ethyl-n-pentyl-phosphonium-bis-(trifluoromethylsulfonyl)amide [P_2225_][TFSA]. While the ionic liquid served as a diluent in the organic phase, the extraction mechanism was consistent with an anionic type, involving the transfer of RuCl_4_^−^ species from the acid chloride aqueous phase. Ruthenium stripping from the loaded extractant was achieved through direct electro-deposition at potentials ranging from −2.5 V to −2.9 V vs. Pt. The resulting deposits contained 86–88% ruthenium, with approximately 4% oxygen and around 2% of fluorine, carbon, and phosphorus. Additionally, the potential for reusing the extractant for continuous extraction and electrolysis was evaluated over ten cycles. Both extraction and current efficiencies gradually declined over successive cycles, from 96% to 57% and from 93% to 87%, respectively. In turn, Rzelewska et al. [90] utilized pure ionic liquids as extractants. Among them, Cyphos IL 167 demonstrated the highest efficiency, with a 70% extraction of Ru(III) from an acid chloride solution, while Cyphos IL 101 and Cyphos IL 104 showed extraction efficiencies of around 50%. Stripping the metal with an acid thiourea solution enabled the recovery of 90% of the metal ions from the organic phase.

Alternatively, Mokhodoeva et al. [91] explored hydrophobic deep eutectic solvents as novel extractants for PGM ions from chloride medium. They used four mixtures of tetraoctylammonium bromide TOAB with carboxylic acids: hexanoic, heptanoic, octanoic, or nonanoic. In all cases, the same level of ruthenium extraction was achieved (86%), which was slightly lower than the results for platinum and palladium (over 98%), but higher than for iridium (34–45%) and rhodium (7–28%). Stripping tests performed on the TOAB-hexanoic acid system with various agents showed the best performance with thiourea-HCl solution, while nitric acid exhibited lower efficiency (75%) and ammonia had no effect. A similar trend was observed for platinum, iridium, and rhodium species. Interestingly, palladium ions were completely stripped by ammonia, while HNO_3_ had no effect.

Shep and Arbad [92] conducted the extractive recovery from an unconventional solutions of organic acids using 2-dodecylaminopyridine. The best performance was achieved for 0.04–0.05 M acid solutions, with the extraction efficiency following the order: oxalic acid < citric acid < succinic acid < malonic acid. The highest recovery of ruthenium(III) of 99.5% was obtained with malonic acid, regardless of the other metal cations (Pt, Pd, Rh, Au, Fe, Ni, Cu) added to the solution. Stripping with 2% NaCl solution showed potential as an environmentally friendly and cost-effective method.

### 6.3. Electrodeposition

**Figure 13:**
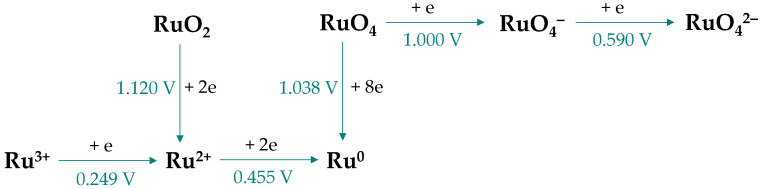
Pathways of reduction ruthenium species in aqueous solutions with standard electrode potentials vs. SHE (based on [9]).

Electrodeposition is one of the methods used during the final stages of metal recovery (electrowinning, electrorefining). While it is commonly applied to aqueous solutions, it can also be carried out with organic electrolytes. However, in the case of ruthenium, deposition is complicated by the possibility of gradual reduction in the ions to lower oxidation states (Figure 13) and depends on their form in the electrolyte as ruthenium ions tend to form various complex species.

Chen et al. [93] showed that the reduction of ruthenium (500 ppm in HCl, platinum substrate) is influenced by the hydrogen evolution reaction. The reduction process varies at different potentials, with reduction currents for Ru(VIII)/Ru and Ru(IV)/Ru(III) observed at 0.88 V and 0.6 V (vs. Ag/AgCl), respectively. The reduction in Ru(III) to Ru started ate potentials more negative than −0.2 V and accompanied by a significant increase in mass ad formation thin and cracked deposits of metallic ruthenium with current efficiencies of only 11–18%. Quite similar deposition potential range was observed in nitric acid solution with two-step reduction Ru(III) to Ru(II) and then to metal [94].

Keal et al. [95] introduced a novel electrochemical nano-impact method for the recovery of ruthenium from highly diluted solutions. This technique, an alternative to traditional galvanostatic or potentiostatic deposition, involves the formation of deposits during the impact of non-metallic nanoparticles onto a working electrode. When the working electrode is held at a sufficiently reducing potential, the particles themselves are directly reduced. The study demonstrated that the electroreduction of Ru(III) species from an acid chloride solution onto carbon black nanoparticles led to the formation of RuO_x_ deposits. This approach achieved over 90% ruthenium recovery from a 1 mM (101 ppm) solution (within about 8 h).

Jayakumar et al. [96] studied the electrodeposition of ruthenium onto stainless steel from aqueous nitric acid-based solution and two ionic liquids (60 ppm Ru^3+^). They found that only 3.7% of ruthenium was recovered from the aqueous electrolyte (4 M HNO_3_, −0.95 V vs. Pd, 8.5 h) with a current efficiency of about 4%. Ruthenium was not reduced on the cathode when using 1-butyl-3-methylimidazolium chloride ionic liquid [BMIM][Cl], even after prolonged electrolysis (30 h, −1.8 V vs. Pd). However, the current efficiency increased to 10% when 1-butyl-3-methylimidazolium hexafluorophosphate ionic liquid [BMIM][PF_6_] was used as the electrolyte (−1.0 V vs. Pd, 3 h).

## 7. Ruthenium Recovery from Spent Nuclear Fuel

Ruthenium is one of the by-products of fission reactions in nuclear reactors. It forms nuclides with significant fission yields (10% for ^235^U fission and up to 40% for ^239^Pu fission) and relatively long half-lives (^103^Ru at 39.3 days, ^106^Ru at 373.6 days) [86]. It is estimated that one tonne of spent fuel with a burn-up of 33 GWd/t contains even 3.2 kg (3222 ppm) of ruthenium [97], about 70% of which is in stable isotope form [98]. This could represent nearly 25 tonnes of the world’s annual production if all discharged spent fuel were reprocessed each year. Furthermore, Burg and Poinssot [97] estimated that the potential global reserves of ruthenium in nuclear waste could reach about 678,600 tons (with a cooling time of 30 years) for the reference year 2014. Thus, the growing interest in ruthenium recovery from spent nuclear fuel becomes understandable (e.g., ^106^Ru isotope for medical applications [98]) [86,99]. Although, in theory, spent fuel could be reused after 10 years of cooling, followed by another 25 years of interim storage (35 years in total) [97], specific separation processes must undoubtedly be developed, although these will likely be complex and expensive to implement in a nuclear environment.

The primary method used for reprocessing spent fuel is the Plutonium Uranium Reduction Extraction (PUREX) process. It involves the dissolution of spent fuel in nitric acid (4–9 M or higher) followed by the solvent extraction of uranium and plutonium using TBP as the first reprocessing steps [100]. Ruthenium species are considered very troublesome nuclides in this operation due to their unpredictable behavior and physicochemical properties (the existence of numerous ruthenium species at different oxidation states, the release of volatile and toxic ruthenium tetroxide, and the potential for decomposition into RuO_2_) [99,101,102]. The primary by-products of the PUREX process are aqueous raffinates from the solvent extraction cycle, which contain highly radioactive fission products, referred to as acidic high-level liquid waste HLLW. This liquid by-product require further treatment, typically through evaporation and vitrification (incorporating into a durable glass matrix) for long-term storage and disposal [100]. In contrast, alkaline intermediate level liquid waste ILLW (pH 12–13) can contain significant amounts of long-lived radionuclides that require isolation and containment for periods greater than several hundred years. HLLW and ILLW represent the main sources for potential ruthenium recovery (Table 4) as these contain ruthenium species at few hundreds ppm levels (or 0.1–0.8 mCi/L ^106^Ru in real ILLW) [103,104,105,106,107,108].

Several recovery methods have been explored for ruthenium extraction, many of which are also commonly applied to other wastewater treatments [86,99]. In the case of HLLW, however, an additional challenge arises from the high concentration of nitric acid, which requires special attention to the process characteristics. Among the various techniques, it is worth highlighting some simple yet effective and unconventional solutions. For example, Li et al. [107] developed a photoreduction process using isopropanol to reduce Ru^3+^ ions species to metallic particles under UV radiation (a mercury lamp). Other alcohols such as methanol, ethanol, and tertiary butanol were ineffective for ruthenium reduction but successfully reduced palladium; thus, the two metals can be easily separated from HLLW by selecting the appropriate reductant (ethanol for palladium, isopropanol for ruthenium), followed by centrifugation of the precipitates. In turn, Davydova and Korolev [105] reported that iron(III) hexacyanoferrate(II) Fe_4_[Fe(CN)_6_]_3_ is a highly efficient adsorbent for Ru(III), Rh(III), and Pd(II) ions from acidic nitric solutions, though it is selective for Pd(II) when acid chloride solutions are used. Swain et al. [108] applied anodic oxidation to convert Ru^3+^ into volatile RuO_4_ via electrolysis in a diaphragm electrolyzer. This process efficiently removed ruthenium from acidic nitrate solution and trapped the volatile tetroxide in a paraffin layer.

## 8. Conclusions

**Figure 14:**
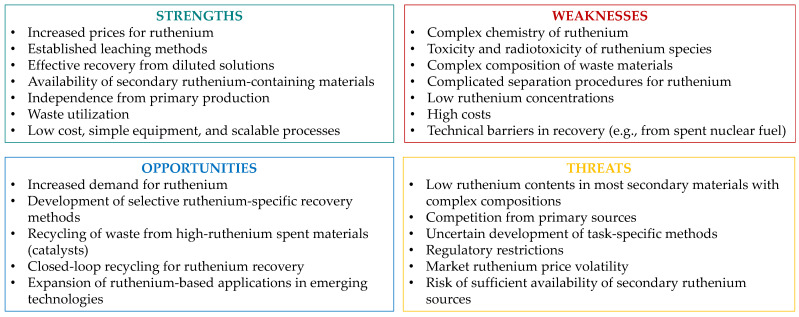
SWOT analysis of ruthenium recovery from secondary materials.

Ruthenium is becoming an increasingly important metal in modern and advanced technological applications. Its production, currently concentrated in one region of the world, is starting to gradually lag behind the growing demand. This creates new opportunities but also challenges for metal recovery from secondary sources (Figure 14). Waste materials not only have higher ruthenium concentrations but also can possess a slightly simpler polymetallic composition compared to natural ruthenium-bearing ores. These factors stimulate scientific advancement and the development of hydrometallurgical processes. There is potential to create sustainable and cost-effective methods, ultimately leading to scalability and industry adoption, despite the limitations imposed by the complex chemistry of ruthenium species and the toxicity of its compounds.

Due to the increasing consumption of ruthenium for chemical catalysis, spent catalysts appear to be the most promising secondary source of ruthenium. This is particularly relevant because their hydrometallurgical recycling can be naturally integrated into existing refinery technologies, which are already well-developed and optimized for the recovery of platinum-group metals. Incorporating spent catalyst recycling into these processes offers the potential for high recovery efficiency, reduced environmental impact, and improved economic viability, making it an attractive strategy for both industrial and sustainable resource management. However, the opportunities offered by innovative methods exploiting the specific properties of ruthenium ions, such as solvent extraction, ion-exchange or adsorption processes, enable the recovery of ruthenium even from highly diluted solutions, which is particularly valuable for the treatment of various wastewater streams. The next crucial step is to identify and characterize these streams, as doing so would allow these methods to fill the existing gap in ruthenium recovery and expand the range of process and wastewater sources that can be efficiently treated.

## Figures and Tables

**Table 1:** Ruthenium concentration in natural sources (ores or sediments and volcanic ash).

Country	Region	Host Materials	Ru Content, ppb	Ref.
Australia	Murphy Well	Komatiitic chromite	220–540	[25]
	Walter Williams Formation, West	Chromite	<150	[18]
	Walter Williams Formation, East		>150	
Canada	Thetford Mines Ophiolite	Chromitite	51–995	[24]
	Sudbury Complex	Ni-Fe sulfides	40	[30]
China	Jinping-Song Da Region, Nanke Deposit	Ni-Cu sulfides	0.1–3.3 (1.2–14.1 in sulfides)	[19]
	Meishan	Sediments and volcanic ash	0.004–0.038	[20]
	Xinmin		0.006–0.039	
Cyprus	Trodos	Chromite	21–160	[28]
Finland	Penikat intrusion	Ni-Fe-Cu sulfides	88–347	[27]
Greece	Orthrys	Chromite	14–110	[28]
	Pindos		15–550	
	Vourinos		32–50	
India	Boula-Nuasahi Area, Odisha	Fe-Cu sulfides	40–171	[22]
		Chromite	23–74	
Iran	Abdasht	Chromite concentrates	7–140	[23]
	Faryab		29–109	
	Neyriz		37–748	
	Sikhuran		4–294	
Japan	Ubara	Sediments and volcanic ash	0.01–0.15	[20]
Philippines	Acoje Block, Zambales	Chromitite	<100–1100	[29]
Russia	Centralonoye I, Polar Urals	Chromite	18–170 (avg. 70)	[24]
	Centralonoye II, Polar Urals		13–260 (avg. 113)	
	Norilsk-Talnakh Mining Region	Pt-Ni-Cu sulfides	10–50	[21]
	Norilsk, Medvezky Creek mine	Ni-Fe-Cu sulfides	170	[27]
South Africa	Bushveld Complex, Merensky Reef	Ni-Fe-Cu sulfides	230–1650	[27,30]
	Bushveld Complex, Platreef	Ni-Fe-Cu sulfides	140	[30]
	Bushveld Complex, UG2 Reef	Chromitite	940–2130	[29]
Turkey	Guleman	Chromite	80	[28]
USA	Stillwater Complex	Chromitite	5–410	[26,29]
Zimbabwe	Great Dyke, Mimosa Mine	Ni-Fe-Cu sulfides	131	[27]

**Table 2:** Ruthenium recovery from simulated (S) and real (R) wastewaters (alkaline ‘al’ or acid ‘ac’).

Waste Source	Ruthenium Concentration, ppm	Recovery Method	Recovery	Ref.
IC manufacturing (S, al)	200	Cementation with Al powder (2 h)	99%	[75]
Ru plating workshop (R, ac)	547	Cementation with Zn powder + NaCl (1 h)	95%	[77]
Chloride solution (S, ac)	20–3000	Adsorption on mineral gel Mg-ACCP (30 s); conversion into RuO_2_	98%	[78]
Ru electroplating (S, ac)	1060	Adsorption on TRPO/SiO_2_-P + NaNO_2_ (3 h)	98%	[79]
Ru electroplating (R, ac)	3250	Adsorption on bacterial biomass (*Rhodopseudomonas palustris*)	32 mg/g	[80]
Ru electroplating (R, al)	1800		38 mg/g	
Chloride solution (S, ac)	100	Adsorption on bacterial biomass (*Pseudomonas aeruginosa*)	196 mg/g	[81]
Acetic acid manufacturing (R, ac)	1823	Adsorption on PEI-modified bacterial biosorbent (*C. glutamicum*) fiber	88 mg/g	[82]
	66.6	Biosorption (*C. glutamicum*-modified PEI fiber), incineration, leaching (NaClO, KMnO_4_, aqua regia), smelting	no data	[83]
	78.2	Adsorption on PEI-PS modified bacterial biosorbent (*Escherichia coli*) fibers	121 mg/g	[84]
	1823	Adsorption on ion-exchange resin (Lewatit MonoPlus M600)	2.5 mg/g	[82]
	78.2	Adsorption on ion-exchange resin (TP214)	60 mg/g	[84]
	78.2	Adsorption on ion-exchange resin (Amberjet 4200)	30 mg/g	

**Table 3:** Ruthenium recovery through solvent extraction.

Solution Type	Ruthenium Concentration, ppm	Extraction/Stripping	Recovery, % Extraction/Stripping	Ref.
Acid, chloride (RuCl_3_ + HCl + NaCl)	100	Aliquat 336 in kerosene (+TBP *)/3 M Na_2_CO_3_	95/84	[88]
Acid, chloride (RuCl_3_ + HCl)	3.8	Aliquat 336 in [P_2225_][TFSA] (+1-decanol *)/Ru electrodeposition	96/93	[89]
Acid, chloride (RuCl_3_ + HCl)	172	Cyphos IL 101/thiourea in HCl	50/93	[90]
		Cyphos IL 104/thiourea in HCl	50/87	
		Cyphos IL 167/thiourea in HCl	70/92	
Acid, chloride (RuCl_3_ + HCl)	10	TOAB + hexanoic acid/thiourea in HCl	87/99	[91]
		TOAB + heptanoic acid/thiourea in HCl	86/no data	
		TOAB + octanoic acid/thiourea in HCl	86/no data	
		TOAB + nonanoic acid/thiourea in HCl	86/no data	
Acid, malonate (RuCl_3_)	100	2-dodecylaminopirydine/2% NaCl	99.5/no data	[92]

* Modifier.

**Table 4:** Ruthenium recovery from simulated (S) or real (R) HLLW and ILLW.

Solution Type	Ruthenium Concentration, ppm	Recovery Method	Recovery, %	Ref.
HLLW (S)	624 ± 31	Solvent extraction: TODGA in n-dodecane	15.3	[103]
		Solvent extraction: TDDGA in n-dodecane	25.8	
		Solvent extraction: DOHyA in n-dodecane	22.9	
HLLW (S)	225	Adsorption on Fe_4_[Fe(CN)_6_]_3_	98	[105]
HLLW (S)	100	Photoreduction: isopropanol + UV	92.5	[107]
HLLW (S)	160	Electrooxidation with RuO_4_ trapping by n-paraffin hydrocarbon	80	[108]
ILLW (R)	0.25 mCi/L	Solvent extraction: Aliquat 336 + 10% isodecyl alcohol in n-dodecane	98	[104]
ILLW (R)	100	Extraction chromatography: Chromosorb W modified with Aliquat 336	82	[106]

## Data Availability

No new data were created or analyzed in this study. Data sharing is not applicable.

## Abbreviations

The following abbreviations are used in this manuscript:

PGM	Platinum group metals
PEI	Polyethylenimine
TBP	Tributyl phosphate
SHE	Standard hydrogen electrode
HLLW	High level liquid waste
ILLW	Intermediate level liquid waste
TDDGA	N,N,N′,N′-tetradecyldiglycolamide
TODGA	N,N,N′,N′-tetraoctyldiglycolamide
DOHyA	N,N-di-octyl-2-hydroxyacetamide
